# Modulation of Cardiac Fibrosis in and Beyond Cells

**DOI:** 10.3389/fmolb.2021.750626

**Published:** 2021-10-27

**Authors:** Dong Fan, Zamaneh Kassiri

**Affiliations:** ^1^ Department of Pathology, Zhuhai Campus of Zunyi Medical University, Zhuhai, China; ^2^ Department of Physiology, Cardiovascular Research Center, University of Alberta, Edmonton, AB, Canada

**Keywords:** fibrosis, tissue inhibitor of metalloproteinases, heart diseases, sex differences, cardiac fibroblast, myocardial infarction

## Abstract

The extracellular matrix (ECM) plays important roles in maintaining physiological structure and functions of various tissues and organs. Cardiac fibrosis is the excess deposition of ECM, including both fibrillar (collagens I and III) and non-fibrillar proteins. Characteristics of fibrosis can vary depending on the pathology, with focal fibrosis occurring following myocardial infarction (MI), and diffuse interstitial and perivascular fibrosis mainly in non-ischemic heart diseases. Compliance of the fibrotic tissue is significantly lower than the normal myocardium, and this can compromise the diastolic, as well as systolic dysfunction. Therefore, strategies to combat cardiac fibrosis have been investigated. Upon injury or inflammation, activated cardiac fibroblasts (myofibroblasts) produce more ECM proteins and cause fibrosis. The activation could be inhibited or the myofibroblasts could be ablated by targeting their specific expressed proteins. Modulation of tissue inhibitors of metalloproteinases (TIMPs) and moderate exercise can also suppress cardiac fibrosis. More recently, sex differences in cardiac fibrosis have come to light with differential fibrotic response in heart diseases as well as in fibroblast functions *in vitro*. This mini-review discusses recent progress in cardiac fibroblasts, TIMPs, sex differences and exercise in modulation of cardiac fibrosis.

## Introduction

Extracellular matrix (ECM) functions as a structural support, an extracellular reservoir, and an interstitial transport system (Fan et al., 2014a; Frangogiannis, 2019). Cardiac ECM proteins are mainly produced by cardiac fibroblasts. Cardiac fibroblasts can be classified into heterogeneous subsets based on location, origin and by using gene expression through single-cell RNA sequencing analysis as reviewed in Tallquist (2020). The ECM can be degraded by matrix metalloproteinases (MMPs) which is controlled by their inhibitors, primarily tissue inhibitors of metalloproteinases (TIMPs), and the balance of MMPs and TIMPs maintained the ECM homeostasis in physiological status (Moore et al., 2012), although MMPs can also contribute to fibrosis (Zile et al., 2014; Takawale et al., 2015). Fibrosis is excess accumulation of fibrillar and non-fibrillar ECM, such as fibrillar collagen types I and III, fibronectin, as along with upregulation of matricellular proteins such as secreted protein acidic and rich in cysteine (SPARC), osteopontin, periostin (Chute et al., 2019; de Boer et al., 2019; Frangogiannis, 2019). Two types of myocardial fibrosis can be identified: the macroscopic focal fibrotic scar, also known as replacement or reparative fibrosis which is observed in myocardial infarction (MI); and diffuse interstitial and perivascular fibrosis that is generally detected in hypertrophic, inflammatory, diabetic cardiomyopathy and the surviving myocardium after MI (Frangogiannis, 2019; Lopez et al., 2021).

Focal fibrosis plays an important role in cardiac repair post-MI due to lack of regenerative capacity of cardiomyocytes, which maintains the structural integrity of the infarcted heart and prevents it from rupture, however if not controlled, its expansion into the non-infarct myocardium can exacerbate post-MI pathology (de Boer et al., 2019; Frangogiannis, 2019). Fibrosis also compromises myocardial compliance and is associated with diastolic dysfunction, contributing to a variety of heart diseases, including heart failure (de Boer et al., 2019; Frangogiannis, 2019). Therefore, the anti-fibrotic treatment is an essential treatment in heart diseases; and some drugs, such as angiotensin-converting enzyme inhibitors, β-blockers, and transforming growth factor β1 (TGFβ1) inhibitors such as pirfenidone, have been applied to directly and/or indirectly target fibrosis (Graziani et al., 2021; Lopez et al., 2021). Recently, targeting activated cardiac fibroblasts and TIMPs have been employed to combat cardiac fibrosis (Purcell et al., 2018; Aghajanian et al., 2019). Fibroblast functions and cardiac fibrosis can be influenced by sex differences (Peter et al., 2021). Sex differences also exist in exercise-associated myocardial fibrosis (Tahir et al., 2018). Therefore, this mini review provides a summary of new targets as potential treatments against fibrosis, and focuses on the roles of cardiac fibroblasts, TIMPs, sex differences, and exercise in modulation of fibrosis in heart diseases.

## Targeting Activated Cardiac Fibroblasts for Anti-Fibrosis

In response to injury or inflammation, cardiac fibroblasts are activated and differentiated into myofibroblasts which increase synthesis and secretion of ECM proteins leading to fibrosis (Khalil et al., 2017; Fu et al., 2018; Aujla and Kassiri, 2021). Activated cardiac fibroblasts (myofibroblasts) express some proteins such as periostin or fibroblast activation protein (FSP) which have little to no expression in quiescent fibroblasts (Kaur et al., 2016; Aghajanian et al., 2019). The FSP was used as a target of engineered chimeric antigen receptor (CAR)-T cells, and the CAR-T cells could specifically eliminate the activated fibroblasts, suppress angiotensin II/phenylephrine-induced cardiac interstitial fibrosis (Table 1), and improve cardiac function (Aghajanian et al., 2019). The ablation of periostin-expressing cardiac fibroblasts with diphtheria toxin in a mouse MI model also reduced excess fibrosis but did not affect the scar thickness or the integrity of the infarct heart, and it increased the proportion and size of cardiomyocytes in the infarct area and improved cardiac function post-MI (Kaur et al., 2016). However, cardiac fibroblasts were found to differentiate into profibrotic myofibroblasts by 3–7 days post-MI, followed by the form of matrifibrocyte which lost proliferative capacity and alpha-smooth muscle actin expression but expressed ECM and tendon genes to support the mature fibrotic scar by 7–10 days post-MI (Fu et al., 2018). A subtype of antifibrotic myofibroblasts were also identified in the day 7 post-MI (Farbehi et al., 2019). Therefore, it is promising to target and eliminate activated cardiac fibroblasts using CAR-T cells, but the effects may be limited to specific diseases and the stage of the disease.

**TABLE 1:** Selected animal model studies suggesting promising therapeutic strategies to combat cardiac fibrosis.

Approaches	Methods	Model	References
Ablating activated cardiac fibroblasts	CAR-T	Ang II/phenylephrine treatment in mice	Aghajanian et al. (2019)
Suppressing activation of cardiac fibroblasts	Macrophage specific deletion of microRNA-21	pressure overload in mice	Ramanujam et al. (2021)
Targeting TGFβ1	TGFβ1-neutralizing antibody (1D11)	pressure overload in mice	Kassiri et al. (2009)
MMP inhibition	PD166793	pressure overload in mice	Kassiri et al. (2005), Kandalam et al. (2011)
	Reduction of MT1-MMP expression	pressure overload in mice	Zile et al. (2014)
TIMPs	Deletion of TIMP1	pressure overload, Ang II treatment in mice	Takawale et al. (2017b)
	TIMP2-contained exosomes	Myocardial infarction in mice	Ni et al. (2019)
	Intracoronary infusion of rTIMP3	ischemia-reperfusion in pigs	Barlow et al. (2017)
	Myocardial injection of MMP-responsive hydrogel releasing rTIMP3	Myocardial infarction in pigs	Purcell et al. (2018)
	Cardiac-restricted overexpression of TIMP4	Pressure overload in mice	Yarbrough et al. (2014)
Exercise	Moderate-intensity exercise training	Aging in mice	Pei et al. (2021)
	Exercise hypertrophic preconditioning	Pressure overload in mice	Lin et al. (2021)

Ang II, angiotensin II; CAR-T, chimeric antigen receptor T-cells; MMP, matrix metalloproteinase; MT1-MMP, membrane type 1-MMP; rTIMP3, recombinant tissue inhibitor of metalloproteinase-3; TGFβ1, transforming growth factor β1.

The activation of cardiac fibroblasts could also be suppressed by macrophage-specific deletion of microRNA-21 which decreased interstitial fibrosis in mice following pressure overload (Ramanujam et al., 2021). Deletion of microRNA-21 in macrophages increased M2-polarized macrophages, while decreased M1-like proinflammatory macrophages which might disrupted the macrophage-to-fibroblast signaling and activation of cardiac fibroblasts (Table 1) (Ramanujam et al., 2021). However, during the proliferative phase of MI, CD226 deletion also increased M2-macrophages and decreased M1-macrophages, but it resulted in accumulation of myofibroblasts and collagen deposition in the infarct area, with reduced interstitial fibrosis in the infarct border area though (Li et al., 2020). Therefore, the regulation of macrophages has different role in the state of cardiac fibroblasts and fibrosis but is beneficial to these two animal models, while the genes deleted were different which might partly cause the difference.

It has also been demonstrated that the activities of myofibroblasts can be terminated during normal tissue repair in the ways such as deactivation, apoptosis and immune clearance of senescent myofibroblasts (Hinz and Lagares, 2020; Merkt et al., 2021). Myofibroblasts Express higher levels of pro-apoptotic molecules such as BCL-2 family member BIM than the quiescent fibroblasts and prime for apoptosis, but they could evade apoptosis by activating pro-survival signals such as TGFβ1 (Piek et al., 2016; Hinz and Lagares, 2020; Merkt et al., 2021). Myofibroblasts can also become senescent, and accumulation of pro-fibrotic senescent myofibroblasts can result in progressive fibrosis. However, senescence can also keep the cells in a cell cycle arrest as a protective mechanism against tumorigenesis through senescence-activating signaling pathways including cyclin-dependent kinase inhibitors p53 and p21 (Hinz and Lagares, 2020). Inhibition of TGFβ1 activation or promotion of apoptosis have been investigated to target myofibroblasts as anti-fibrotic strategies, especially in lung fibrosis and skin scarring (Hinz and Lagares, 2020).

## TIMPs and Cardiac Fibrosis

Besides secreting ECM proteins, cardiac fibroblasts can produce ECM degrading enzymes (MMPs) and their inhibitors, TIMPs (TIMP1, TIMP2, TIMP3, and TIMP4) (Fan et al., 2012; Takawale et al., 2015). MMPs, a family of zinc-dependent proteolytic enzymes, can be separated into six groups according to their structure and substrates, including collagenases (MMP1, −8, −13), gelatinases (MMP2, −9), stromelysins (MMP3, −10, −11), metrilysins (MMP7, −26), membrane type MMPs (MT-MMPs, MMP14, −15, −16, −24, −17, and −25), and other MMPs (Takawale et al., 2015). TIMPs can bind and inhibited activated MMPs, but TIMP2 can also bind to proMMP2 and form the MT1-MMP-TIMP2-proMMP2 complex which assists the cleavage and activation of proMMP2 by a second MT1-MMP at the cell surface (Strongin et al., 1995; Jackson et al., 2017). TIMPs can also interact with other proteins and molecules to regulate cardiac fibrosis, hypertrophy, and angiogenesis (Moore et al., 2012; Takawale et al., 2015; Jackson et al., 2017).

### TIMP1 as an Anti-Fibrosis Target

An increase in TIMP1 expression has been consistently linked to cardiac fibrosis (Moore et al., 2012). In the heart, TIMP1 can bind to its receptor CD63 and mediate its association with integrin β1, followed by activation of Smad2/3 and β-catenin, and subsequently promoting collagen production and cardiac fibrosis (Takawale et al., 2017b). Deletion of TIMP1 suppressed mouse myocardial fibrosis induced by transverse aortic constriction (TAC) or angiotensin II (Table 1), which might be caused by disrupting the interaction of CD63 and integrin β1 (Takawale et al., 2017b). Therefore, deletion of TIMP1 showed anti-fibrotic effect in these two mouse models, which remains to be confirmed in large animal models and in humans.

### TIMP2 and Cardiac Fibrosis

TIMP2 is a potent inhibitor of MT1-MMP, a well-known collagenase that is upregulated in heart disease (Takawale et al., 2014). Loss of TIMP2 (*Timp2* ^−/−^) leads to severe myocardial interstitial fibrosis and disorganization of ECM in mice following pressure overload compared to wild-type (WT) mice (Kandalam et al., 2011). These mice exhibited non-uniform ECM remodeling with increased ECM degradation in some areas and excess fibrillar deposition in other areas of the myocardium despite comparable mRNA expression of collagen I and III compared to parallel WT mice (Kandalam et al., 2011). The increased collagen degradation could be caused by higher collagenase activities, especially MT1-MMP, in the absence of TIMP2 (Kandalam et al., 2011). Interestingly, despite its strong collagenase activities, MT1-MMP overexpression has been reported to promote cardiac fibrosis through activation of TGFβ by mediating its proteolytic release from its latent form in the ECM (Zile et al., 2014). Therefore, the contribution of MT1-MMP to fibrosis appears to be a balance between its collagen-degrading and TGFβ-activating functions. General inhibition of MMPs with PD166793 decreased the total collagenase activity, as well as MT1-MMP activity, and reduced cardiac interstitial fibrosis in *Timp2* ^−/−^-TAC mice (Table 1) (Kandalam et al., 2011), which is consistent with the finding that reduction of MT1-MMP expression suppressed cardiac fibrosis and improved cardiac function in mice after pressure overload (Table 1) (Zile et al., 2014). In contrast to the pro-fibrosis effects of TIMP2-deficiency in response to the mechanical stress induced by pressure overload, pharmacological induction of fibrosis by angiotensin II infusion resulted in suppressed cardiac fibrosis in *Timp2* ^−/−^ mice, which was found to be linked to decreased collagen cross-linking proteins PLOD1 and LOX (Fan et al., 2014b). Therefore, the role of TIMP2 in cardiac fibrosis depends on the type of injury or stimulus.

In a mouse MI model, lack of TIMP2 resulted in augmented degradation and disorganization of collagen fibers, which might be caused by elevated total collagenase activity (mainly MT1-MMP) (Kandalam et al., 2010b). Exosomes derived from TIMP2-overexpressing human umbilical cord mesenchymal stem cells can reduce both myocardial fibrosis and apoptosis of myocytes, and enhance angiogenesis in mice after 30 days post-MI (Ni et al., 2019). The TIMP2-contained exosomes could inhibit the proliferation and activation of cardiac fibroblasts (Ni et al., 2019), which may mediate its anti-fibrotic effects. However, local myocardial injection of adenovirus expressing TIMP2 had no significant effect on cardiac fibrosis following MI; although it suppressed the expression and activity of MMP2 and MMP9, infiltration of inflammatory cells, and improved the survival rate and cardiac function to some extent up to 7 days post-MI (Ramani et al., 2011). It is worthy to investigate whether overexpression of TIMP2 could counter fibrosis expansion post-MI over long-term.

### TIMP3 and Anti-Fibrosis Effects

TIMP3 has a wide range of inhibitive substrates and interactive molecules (Moore et al., 2012; Fan and Kassiri, 2020). Deficiency of TIMP3 led to extensive cardiac fibrosis through activation of TNF-α, TGFβ1 and downstream molecules Smad2/3 after pressure overload in mice, which could be inhibited by a TGFβ1 neutralizing antibody (Table 1) (Kassiri et al., 2005; Kassiri et al., 2009). Deletion of TIMP3 also exacerbated myocardial fibrosis induced by angiotensin II (Fan et al., 2014b). The excessive cardiac fibrosis is caused by stabilization of collagen fibers due to increased SPARC and osteopontin, without significant changes in collagen *de novo* synthesis (Fan et al., 2014b). However, deficiency of TIMP3 reduced collagen density following MI, leading to increased cardiac rupture and mortality which was reduced by inhibition of total MMPs (Kandalam et al., 2010a; Hammoud et al., 2011). Therefore, lack of TIMP3 has different roles in cardiac fibrosis in different animal models.

Overexpression of TIMP3 in infarct myocardium suppressed MMP activity, degradation and disorganization of collagen fibers, while also promoting angiogenesis post-MI (Takawale et al., 2017a). As MMP activities increase following MI, intracoronary- and MMP-degradable hydrogel-mediated supplementation of TIMP3 protein suppressed the activation of fibroblasts and cardiac fibrosis in pig ischemia/reperfusion (I/R) or MI models, and improved cardiac function in both models (Table 1) (Barlow et al., 2017; Purcell et al., 2018). On the other hand, the N-terminus of TIMP3 molecule with preserved MMP-inhibitory activity did not affect the collagen accumulation in a pig MI model, but the full-length TIMP3 reduced myocardial fibrosis (Lobb et al., 2020). Therefore, MMP-independent functions of TIMP3 are involved in its anti-fibrotic functions in the heart. A noninvasive imaging approach with single-photon emission computed tomography (SPECT)/CT is also explored in a pig I/R model to monitor the activation of MMPs, myocardial blood flow and strain (Thorn et al., 2019). It facilitates the targeted and timely application of TIMP3 in I/R or MI models and observation of therapeutic effects.

### TIMP4 and Cardiac Fibrosis

TIMP4 is expressed in a variety of organs and highly expressed in the brain and heart (Nuttall et al., 2004). Loss of TIMP4 had no significant effect on descending aortic binding-induced cardiac fibrosis in male mice, but enhanced cardiac fibrosis in a TAC model with both sex which could be reduced by overexpression of TIMP4 (Table 1) (Koskivirta et al., 2010; Yarbrough et al., 2014), suggesting an anti-fibrotic function for TIMP4. On the other hand, lack of TIMP4 decreased the intensity of collagen fibers and the survival rate following MI which could be alleviated by inhibition of MMPs or overexpression of TIMP4 in the mouse MI model (Koskivirta et al., 2010; Zavadzkas et al., 2014). However, deficiency of TIMP4 enhanced myocardial fibrosis in the I/R model which was linked to increased MT1-MMP activity and activation of TGFβ signaling (Takawale et al., 2014). Therefore, the roles of TIMP4 in cardiac fibrosis may vary with animal models and the sex. Recent reports also demonstrated that there were sex differences in cardiac fibrosis which are discussed in the following section.

## Sex Differences in Fibroblast Function and Cardiac Fibrosis

Male patients with severe aortic stenosis awaiting valve replacement had more cardiac fibrosis detected by late gadolinium enhancement (LGE)+ and indexed matrix volume in cardiac magnetic resonance (CMR) imaging and lower left ventricle ejection fraction than female patients (Treibel et al., 2018). However, it has also been reported that female patients with mild to severe aortic stenosis had more diffuse but similar focal cardiac fibrosis (LGE) compared to male patients (Tastet et al., 2020). The difference between these two studies may be caused by the different severity of aortic stenosis in the patient population. Female patients with hypertrophic cardiomyopathy also exhibited more cardiac fibrosis detected by Picro-Sirius Red staining in cardiac tissue obtained during myectomy or heart transplantation than male patients (Nijenkamp et al., 2020). Therefore, there are some discrepancy in cardiac fibrosis reports between male and female patients which needs more comparative investigations.

In animal models, there was no significant difference in cardiac fibrosis between female and male mice after acute (single dose of isoproterenol 10 mg/kg) or chronic adrenergic stimulation (infusion of isoproterenol 10 mg/kg/day for 14 days) (Grant et al., 2020). However, a recent study found that male rats developed cardiac fibrosis in response to isoproterenol stimulation (4 mg/kg/day) while the females did not (Peter et al., 2021). The male rats also exhibited severer cardiac hypertrophy and mortality than the females. Gonadectomy did not change cardiac fibrotic response in both sex of rats in response to isoproterenol treatment, therefore, the sex hormones did not appear to be the determinants of cardiac fibrosis although ovariectomized female rats with isoproterenol had increased mortality (Peter et al., 2021). Interestingly, the cardiac fibroblasts from male rats were found to have higher levels of adrenergic receptors β1 and β2 than the female fibroblasts, which could be responsible for a stronger activation of male cardiac fibroblasts in response to isoproterenol stimulation compared to female fibroblasts (Peter et al., 2021). Therefore, cardiac fibroblasts from different sexes may respond to injuries or stimulation differently, and the sex-related differences in cardiac fibrosis induced by isoproterenol may also vary with species and doses which demand further comparative studies. In an aging model, cardiac fibrosis exhibits different patterns between female and male mice (Achkar et al., 2020). More interstitial fibrosis, but less perivascular and replacement fibrosis were detected in aged female compared to aged male hearts (Achkar et al., 2020). It was further found that collagen III is predominant in female hearts but collagen I in male hearts (Achkar et al., 2020). Therefore, targeting cardiac fibrosis in the aging animal model or patients should be approached with consideration to these sex-dependent differences.

## Exercise and Myocardial Fibrosis

The sex difference also exists in exercise-related cardiac fibrosis. Endurance exercise was found to be associated with myocardial fibrosis based on LGE+ in CMR imaging in male triathletes aged 45 ± 10 years, but not in female athletes (Tahir et al., 2018; Tahir et al., 2020). The triathletes with myocardial focal fibrosis (LGE+) were also older than LGE-athletes (Tahir et al., 2018; Tahir et al., 2020). However, even in young athletes, high blood pressure response to exercise is associated with higher incidence of developing hypertension (Caselli et al., 2019). But lifelong exercise has been found capable to reduce cardiac stiffness in human (Carrick-Ranson et al., 2019). A recent animal study found that moderate-intensity exercise training but not high-intensity interval training reduced cardiac fibrosis (Table 1) and oxidative stress, improved cardiac function in aged (32-week-old) male mice (Pei et al., 2021). Exercise hypertrophic preconditioning in mice could also alleviate cardiac fibrosis (Table 1), hypertrophy and dysfunction at 1 week and 4 weeks post-TAC (Lin et al., 2021). Therefore, moderate exercise can decrease cardiac fibrosis, but endurance exercise can induce cardiac fibrosis to some extent which may be related to the sex, age, and the exercise intensity.

## Future Perspectives and Conclusion

Cardiac fibrosis is an adverse pathological process leading to cardiac dysfunction and heart failure, except some protective role in the infarct heart. Combating myocardial fibrosis can be achieved by inhibiting the activation of cardiac fibroblasts (myofibroblasts), modulation of TIMPs, and moderate exercise. However, the activated cardiac fibroblasts have different subtypes and vary with different heart diseases and disease stages which may hamper the antifibrotic effect by targeting these cells and it needs further investigation in a long term and in human disease. Supplementation of TIMPs has multiple benefits including modulation of fibrosis, promoting angiogenesis and suppressing inflammatory response in animal MI models. Despite many promising experimental discoveries, currently no clinical anti-fibrosis treatment is available. Importantly, sex differences in different types and stages of heart diseases should also be considered in developing anti-fibrosis therapies.
